# Design and Outcomes of a Novel Multidisciplinary Ophthalmic Genetics Clinic

**DOI:** 10.3390/genes14030726

**Published:** 2023-03-15

**Authors:** Bela Parekh, Adelyn Beil, Bridget Blevins, Adam Jacobson, Pamela Williams, Jeffrey W. Innis, Amanda Barone Pritchard, Lev Prasov

**Affiliations:** 1 University of Michigan Medical School, Ann Arbor, MI 48109, USA; 2 Kellogg Eye Center, Department of Ophthalmology and Visual Sciences, University of Michigan, Ann Arbor, MI 48105, USA; 3 Department of Pediatrics, University of Michigan, Ann Arbor, MI 48109, USA; 4 Department of Human Genetics, University of Michigan, Ann Arbor, MI 48109, USA

**Keywords:** ophthalmic genetics, medical genetics, inherited ocular disorders, microphthalmia, congenital cataracts, optic neuropathy, anterior segment dysgenesis, Bosch–Boonstra–Schaaf syndrome, nystagmus

## Abstract

The Multidisciplinary Ophthalmic Genetics Clinic (MOGC) at the University of Michigan Kellogg Eye Center aims to provide medical and ophthalmic genetics care to patients with inherited ocular conditions. We have developed a clinical and referral workflow where each patient undergoes coordinated evaluation by our multidisciplinary team followed by discussions on diagnosis, prognosis, and genetic testing. Testing approaches are specific to each patient and can be targeted (single-gene, gene panel), broad (chromosomal microarray, whole-exome sequencing), or a combination. We hypothesize that this clinic model improves patient outcomes and quality of care. A retrospective chart review of patients in the MOGC from July 2020 to October 2022 revealed that the most common referral diagnoses were congenital cataracts, optic neuropathy, and microphthalmia, with 52% syndromic cases. Within this patient cohort, we saw a 76% uptake for genetic testing, among which 33% received a diagnostic test result. Our results support a tailored approach to genetic testing for specific conditions. Through case examples, we highlight the power and impact of our clinic. By integrating ophthalmic care with medical genetics and counseling, the MOGC has not only helped solve individual patient diagnostic challenges but has aided the greater population in novel genetic discoveries and research towards targeted therapeutics.

## 1. Introduction

Patients affected by ocular genetic conditions often require complex care that is beyond the scope of an ophthalmologist’s role alone [1]. Substantial genetic diagnostic testing, familial counseling, systemic disease monitoring, and low-vision guidance need to be completed additionally [1]. Inaccurate referral diagnoses are still relatively common in 30–50% of cases, highlighting the importance of accurate eye exams and appropriate testing [2,3]. Overlooking ophthalmic features of genetic syndromes can be detrimental given that genetic disease is the most common cause of blindness in young children in developed countries, comprising 50% of all childhood blindness [1]. For example, Cardiac Urogenital Syndrome (CUGS), a genetic condition caused by pathogenic variants in the *MYRF* gene, was initially characterized by congenital diaphragmatic hernia, congenital heart defects, and urogenital defects without clear ocular differences noted [4,5,6,7]. Subsequent reports of familial nanophthalmos were noted to be caused by pathogenic variants in this same gene, and it is now known that the ocular features of CUGS are among the most penetrant and one of the most treatable features of this syndromic condition [8,9,10,11]. Similarly, systemic features can be overlooked in conditions that are thought to cause just ophthalmic abnormalities. For example, a family with a clinical diagnosis of Juvenile Open-Angle Glaucoma (JOAG) was identified to have a pathogenic *DDX58* variant with the proband’s father exhibiting systemic features of Singleton–Merten syndrome, including psoriasiform skin rash, arthritis, spontaneous tendon rupture, and vascular calcifications, in addition to the ophthalmic findings [12]. This overarching diagnosis allowed for targeted therapeutics to be started to treat these symptoms, as *DDX58*-related disease represents a Type I interferonopathy [12].

Much of the care for patients with inherited eye disorders is typically delivered in an uncoordinated manner by different health care providers, who may even work at different hospitals [1]. These silos within the system can lead to the duplication of efforts and a less holistic approach to patient care, leading to additional burdens for patients and their families [1]. In addition, an inaccurate ophthalmic diagnosis can impact the medical geneticist’s evaluation, as correct phenotyping is critical for ordering and interpreting genetic testing [13]. The creation of multidisciplinary ophthalmic and genetics clinics is an effort to address these potential barriers to provide efficient care for patients with inherited ocular disease and genetic syndromes with ocular features. To date, few of these clinics have been described [1,2,13].

Here, we characterize the multidisciplinary ophthalmic genetics clinic (MOGC) at the University of Michigan Kellogg Eye Center, started in July 2020 as a complementary program to an existing retinal dystrophy clinic. We highlight a novel clinic model featuring integrated care with an ophthalmic geneticist, medical geneticist, and genetic counselor. We share successful case examples that demonstrate the value of this care team, and evaluate diagnostic accuracy and genetic testing patterns within our patient population. These results illustrate the advantages of a multidisciplinary approach to patients with inherited ocular conditions.

## 2. Materials and Methods

A cohort study using data from a retrospective chart review of patients referred to the Multidisciplinary Ophthalmic Genetics Clinic at the University of Michigan Kellogg Eye Center between July 2020 and October 2022 was performed (n = 71). Clinical data including the following were collected: prenatal/birth/developmental history, three-generation pedigree, comprehensive physical exam findings (including ocular and systemic), diagnostic radiology imaging, biochemical tests, and genetic testing results. Where indicated, ophthalmic imaging, visual field testing, and electrophysiologic testing were performed, including spectral domain optical coherence tomography (Heidelberg Spectralis, Franklin, MA, USA), Optos wide-field autofluorescence and color imaging (Dunfermline, UK), and Bscan or ultrasound biomicroscopy (Ellex EyeCubed, Minneapolis, MN, USA). Genetic testing approaches, uptake, and results were evaluated for all patients with ocular findings who had been evaluated in the clinic (n = 71). Diagnostic rate was evaluated using established American College of Medical Genetics criteria, and compared to other published panel-based testing utilized by other practices, including the Oculome test, Cat-Map testing for cataracts, and anterior segment dysgenesis panel testing [14,15,16,17].

## 3. Results

### 3.1. The Multidisciplinary Ophthalmic Genetics Clinic

The Multidisciplinary Ophthalmic Genetics Clinic (MOGC) at Kellogg was created to provide medical and ophthalmic genetics care to patients with inherited ocular conditions. MOGC focuses on syndromic and other ocular conditions for both pediatric and adult patients, and is complementary to the existing retinal dystrophy clinic at the Kellogg Eye Center, whose scope centers more on nonsyndromic retinal conditions such as retinitis pigmentosa, cone and cone–rod dystrophy, Stargardt disease, and macular dystrophy.

**Figure 1:**
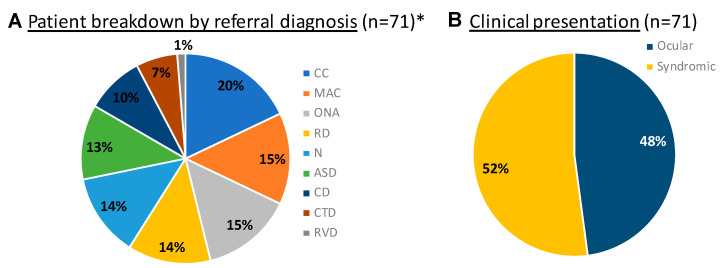
Referral patterns in the clinic. (**A**) Breakdown of the referral diagnoses for the 71 patients seen at the MOGC between July 2020 and October 2022. Categories are as follows: ASD (anterior-segment dysgenesis) including aniridia; Axenfeld–Rieger syndrome, Peters anomaly; CC (congenital or juvenile cataract); CD (corneal dystrophy) including Schnyder crystalline dystrophy, posterior polymorphous corneal dystrophy; CTD (connective tissue disorder), i.e., Stickler syndrome, osteogenesis imperfecta, and ectopia lentis; MAC (microphthalmia/anophthalmia/coloboma); N (nystagmus), including a broad set of diagnoses, i.e., albinism, Leber Congenital Amaurosis, optic neuropathy, and foveal hypoplasia; ONA (optic nerve anomaly/atrophy), i.e., optic nerve hypoplasia and morning glory anomaly; RD (retinal degeneration), including macular dystrophy, cone–rod dystrophy, and retinitis pigmentosa; RVD (retinal vascular disorder), including persistent hyperplasia of primary vitreous and familial exudative vitreoretinopathy. * Note percentages sum up to above 100% as some patients were referred for more than one diagnosis. (**B**) Fraction of patients with purely ocular vs. syndromic presentations. More than half of the patients (52%) seen in the MOGC had a syndromic presentation at their initial visit.

**Figure 2:**
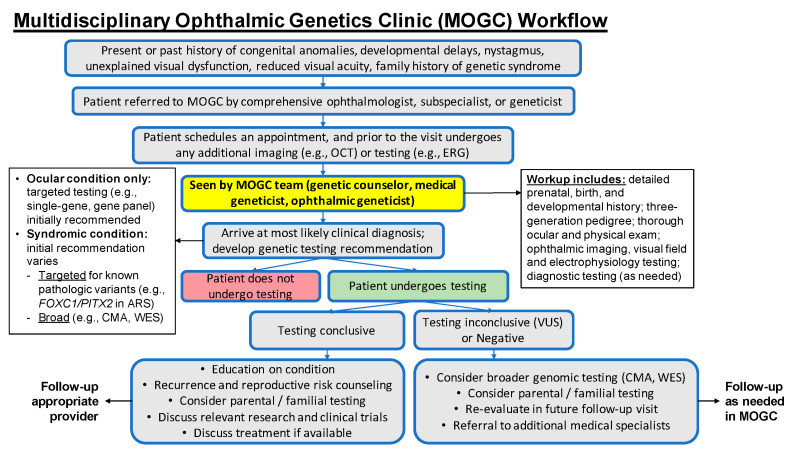
Diagram demonstrating multidisciplinary ophthalmic genetics clinic workflow from referral to follow-up. OCT, optical coherence tomography; ERG, electroretinography; ARS, Axenfeld–Rieger Syndrome; CMA, chromosomal microarray; WES, whole-exome sequencing.

Initially, the clinic started as a hybrid virtual/in-person model (with the ophthalmology exam and testing in clinic and the medical genetics and genetic counseling delivered virtually) during the COVID-19 pandemic and transitioned to a fully in-person monthly clinic as of January 2022. The clinic currently sees referrals primarily from ophthalmologists for a wide variety of inherited ocular diseases and systemic genetic diseases featuring ocular involvement including hereditary optic neuropathy, congenital cataracts, microphthalmia, coloboma, anterior-segment dysgenesis, albinism, and syndromic retinal dystrophy (Figure 1). Our core clinical team consists of an ophthalmic geneticist, a medical geneticist, and a genetic counselor, with support from a clinic scheduler, ophthalmic technicians, and an electrophysiologist. We developed a clinical and referral workflow (Figure 2), which depicts our decision trees for referral and genetic testing. The MOGC also has the advantage of seeing patients with systemic signs and symptoms (Figure 2), suggesting a possibly syndromic condition (i.e., Stickler syndrome). Each patient undergoes coordinated evaluation by our ophthalmic genetics team followed by discussions on diagnosis, prognosis, and genetic testing. Below, we highlight several familial cases that demonstrate the utility of the multidisciplinary approach.

### 3.2. Approaches to Genetic Testing and Diagnostic Yield

**Figure 3:**
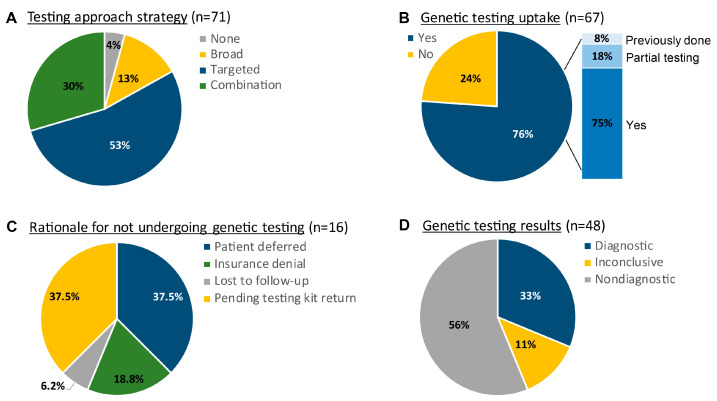
Genetic testing outcomes and strategies. (**A**) Patient testing approach categorized as follows: Targeted—single-gene, gene panel; Broad—chromosomal microarray (CMA), whole-exome sequencing (WES); Combination—both targeted and broad testing recommended. Patients for whom there was no testing approach recommended (“None”) were cases in which no genetic testing was clinically indicated (n = 3). (**B**) Of the patients who were given a recommendation to undergo genetic testing via one of the approaches highlighted above (n = 67)—e.g., targeted, broad, combination—76% (n = 51/67) moved forward. Among these patients, “previously done” refers to the patients who had undergone complete genetic testing prior to coming to the MOGC; “partial testing” refers to patients who completed a portion of recommended testing; “yes” refers to those patients who completed all the recommended testing. (**C**) Patients who did not move forward with genetic testing (n = 16) had a variety of reasons that ranged from personal barriers (“deferred”, “lost to follow up”, “pending testing kit return”) to systemic barriers (“insurance denial”). (**D**) Of the patients who underwent all recommended genetic testing and whose results are not still pending (n = 48), the MOGC demonstrated a diagnostic yield of 33% (n = 16/48).

The scope of genetic testing can be as targeted as single gene panels to as broad as whole-exome sequencing. The MOGC team works to balance the practical considerations of testing, including financial implications and turnaround time, with diagnostic capacity. The result is a varied testing strategy that is patient-specific (Figure 3). Within this patient cohort, we saw a 76% uptake in genetic testing, if recommended, among which there were diagnostic results in 33% of cases (Figure 3). Diagnostic yield in the MOGC for specific referral conditions varied, with the greatest success seen in anterior-segment dysgenesis patients (Table 1). For those patients who chose not to undergo genetic testing, the reasons included both personal (i.e., patient deferral) and financial (i.e., insurance denial), though a top reason also included a logistical barrier (i.e., returning DNA testing kits) (Figure 3).

### 3.3. Case Reports

#### 3.3.1. Syndromic Optic Neuropathy

**Figure 4:**
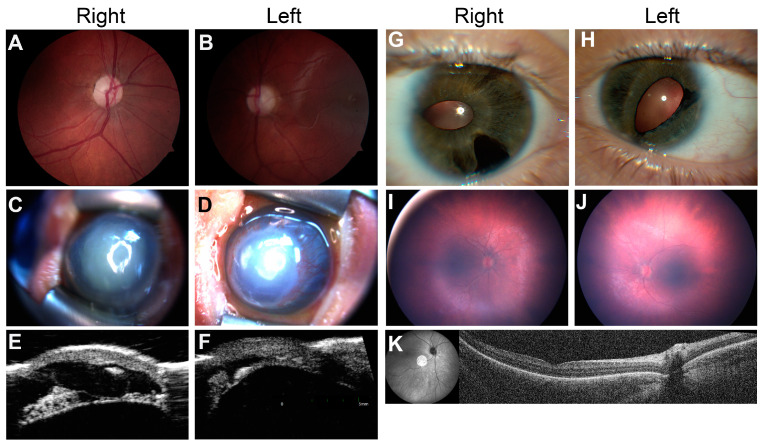
Clinical imaging in highlighted cases (**A**,**B**) Case 1 fundus exam photographs demonstrating optic disc pallor and moderate cupping bilaterally. (**C**–**H**) Case 2; (**C**,**D**) External photographs demonstrating an enlarged and thinned cornea, central descemetocele, and peripheral neovascularization. Poorly visualized are aniridia and posterior embryotoxon. (**E**,**F**) Anterior-segment UBM showing a cystic iris stump (**E**) and peripheral synechiae (**F**), consistent with Axenfeld–Rieger syndrome. (**G**,**H**) Proband’s mother’s corectopia and iris atrophy bilaterally. Additionally, a Descemet tear is appreciated in the right eye (**G**). (**I**–**K**) Case 3 (**I**,**J**) RetCam photos showing granular pigment changes and vascular attenuation in both eyes, with decreased foveal light reflex. (**K**) OCT imaging of the right eye showing poor definition of the ellipsoid zone as well as thinning of the outer nuclear layer in the right eye.

An 11-year-old male presented to our clinic with a history of visual impairment, nystagmus, and esotropia with abduction deficits. His vision loss had been grossly stable until the age of presentation and he had previously tried vision therapy for two years with no significant improvement. His past medical history was significant for developmental delay requiring interventions (speech–language pathology, physical and occupational therapy, and an individual education plan in school). He had a history of febrile seizures between 18 months and 5 years of age that were subsequently resolved. In the clinic, his ocular exam was notable for an incomitant esotropia, bilateral abduction deficits, latent nystagmus, and poor visual acuity bilaterally (VA 20/200 OD 20/250-300 OS). A slit-lamp exam showed mild ptosis with an unremarkable anterior-segment exam and normal intraocular pressure (IOP). A fundus exam revealed bilateral loss of foveal light reflex, optic disc pallor, and moderate cupping (Figure 4A,B). A physical exam showed mild dysmorphic features (low-set ears, anteriorly displaced upper teeth, slight upslant to palpebral fissures, micrognathia), hypotonia, and mild gait instability. There was no significant family history of any relevant ocular or systemic conditions.

The patient was diagnosed with syndromic optic atrophy and subsequently underwent genetic testing. Chromosomal microarray testing was negative, and whole-exome sequencing revealed a single de novo pathogenic variant in the *NR2F1* c.90_99del (p.Arg31Alafs*85) associated with Bosch–Boonstra–Schaaf Optic Atrophy Syndrome (BBSOAS), an autosomal dominant condition [18,19]. The haploinsufficiency of NR2F1, a transcription factor for neural development, can lead to several key features of BBSOAS—developmental delay, autism-spectrum disorder, and seizures—and ocular findings such as optic atrophy/hypoplasia and cortical vision loss consistent with our patient’s phenotype [19]. Identifying this diagnosis led to several positive benefits for the patient and his family. First, the constellation of seemingly unrelated symptoms now fit within the context of one diagnosis, and a clear explanation for his ocular and systemic features was identified. Second, he was able to follow up with the low-vision clinic, establish care with a BBSOAS specialist for further clinical care, and even join the BBSOAS patient foundation. Third, his parents learned that this was a de novo variant with low risk of recurrence for future pregnancies. Fourth, the patient was evaluated for other ocular features of BBSOAS such as alacrimia.

#### 3.3.2. Familial Anterior Segment Dysgenesis

The patient was born via cesarean section at 36 weeks and 5 days due to maternal cholestasis. Neonatal intensive care unit physicians noted bilateral absent red reflex and transferred the patient to the University of Michigan for further evaluation. On day 1 of life, a bedside exam showed bilateral corneal opacification and central descemetoceles. A limited view into the anterior chamber at that time showed bilateral aniridia and prominent posterior embryotoxon. The IOP measurement was inaccurate due to the abnormal cornea, but was significantly elevated by palpation, and the patient was started on dorzolamide-timolol and latanoprost. On day 4 of life, the patient was scheduled for an exam under anesthesia with a possible surgical IOP-lowering intervention. The eye was found to be too thin and small for safe incisional surgery, and transscleral contact cyclophotocoagulation was performed.

She was subsequently referred at 5 months of age to the MOGC for genetic evaluation for her severe anterior-segment dysgenesis and secondary glaucoma. She was confirmed to have microphthalmia, bilateral partial aniridia, and bilateral congenital glaucoma in the setting of ongoing growth delays, feeding difficulties, hypotonia, and atrial septal defect. On ocular exam, the patient was aversive to light and found to have horizontal pendular nystagmus. IOP in the right eye was mildly elevated to 22 mmHg, and normal in the left eye (18 mmHg). A slit-lamp exam showed lagophthalmos with corneal thinning and protrusion OU, diffuse corneal haze with overlying keratinization and posterior embryotoxon OU, and peripherally shallow anterior chamber OU (Figure 4C,D). There was no view of the fundus. Ultrasound biomicroscopy (UBM) demonstrated numerous iris cysts in the right eye, with a hypoplastic iris seen in the left eye (Figure 4E,F). A physical exam showed mild dysmorphic features (low-set and posteriorly rotated ears, midfacial hypoplasia, deep palmar creases) and hypotonia. Growth parameters were notable for 12th percentile head circumference (corrected for gestational age), 2nd percentile weight, and <1st percentile for length.

Notably, the patient’s 24-year-old mother had a clinical diagnosis of Axenfeld–Rieger syndrome based on signs of anterior-segment dysgenesis along with a history of dental caries, but she had never undergone prior genetic testing. The mother had a history of glaucoma and extraocular muscle surgery, and was not taking any topical glaucoma medications at the time of data collection. An ophthalmic evaluation of the patient’s mother showed corectopia, iris atrophy, and Descemet tears in the right eye (Figure 4G,H), along with a normal foveal contour on optical coherence tomography and fundus exam. The proband’s 3-year-old maternal half-brother additionally had a history of a corneal ulcer of unknown etiology and speech delay.

Family history and the patient’s clinical presentation were suggestive of *FOXC1*-related anterior-segment-dysgenesis syndrome given the systemic findings, along with congenital glaucoma and normal foveal architecture. She underwent panel-based testing, which revealed a pathogenic *FOXC1* c.135dup (p.V46Rfs*37) variant, inherited from her less severely affected mother. Given the severity of the proband’s phenotype, broad testing with chromosomal microarray and exome sequencing was conducted to rule out a dual diagnosis. This did not reveal any additional pathogenic variants, suggesting the *FOXC1* variant was the sole genetic cause of the patient’s disorder. The family was counseled that *FOXC1* can be associated with hearing loss, brain anomalies, hypotonia, and failure to thrive in addition to ocular anomalies that would require continuous monitoring [15]. Heterozygous variants such as the one identified in both the patient and the patient’s mother have been associated with a spectrum of anterior-eye-segment defects, including Axenfeld–Rieger syndrome [15]. This genetic diagnosis highlighted the spectrum of the anterior-segment-dysgenesis disorders, and the value of testing and counseling for family planning.

#### 3.3.3. Infantile Nystagmus Secondary to Retinal Disorder

A 6-month-old male with an unexplained history of nystagmus and congenital esotropia presented to the MOGC after referral from pediatric ophthalmology. His mother reported that shortly after birth, the patient had intermittent episodes of ocular shaking in both horizontal and vertical directions, worse when the patient was agitated. Pregnancy was uncomplicated and development was otherwise normal. The patient had no known family history of ocular deficits or syndromic conditions. He was given glasses for high hyperopia which seemed to improve, but not correct, visual behavior and strabismus. The slit-lamp exam was unremarkable; however, a fundus exam showed bilateral blunted foveal light reflex (FR), mild vascular attenuation, and granular pigment changes with few areas of pigment clumping (Figure 4I,J). OCT showed a poor definition of ellipsoid zone (EZ) in both eyes (Figure 4K), and electroretinography (ERG) showed evidence of severe reductions in both scotopic and photopic function. Together, these results suggested a severe retinal degeneration consistent with a diagnosis of Leber congenital amaurosis (LCA) [20]. The patient underwent genetic testing with a Blueprint Genetics Retinal Dystrophy panel, which uncovered two likely pathogenic variants in *RPGRIP1* c.1437_1440dup, p.(Val481Serfs*13) and c.3652G>T, p.(Glu1218*), which were consistent with his clinical diagnosis. Segregation analysis revealed that each parent contributed one disease-causing allele, confirming the diagnosis of *RPGRIP1*-related LCA.

Evaluation and diagnosis in the MOGC provided a number of benefits for the patient and his family. First, functional testing and detailed retinal evaluation narrowed the diagnosis of his vision loss to LCA from a wide differential diagnosis for nystagmus and visual impairment (including albinism, optic neuropathy, foveal hypoplasia, congenital nystagmus, and retinal dystrophy), and provided more information about the prognosis and visual function of the patient. Second, he began treatment with orientation specialists, started occupational therapy, and worked with a developmental therapist due to his low vision. He was also equipped with braille reading material. Third, his parents learned about their risk for future pregnancies given the genetic nature of the patient’s condition, and were informed of the patient’s own reproductive risks, as all of his children would be carriers. Fourth, while there is no treatment approved at this time, the family is now exploring investigative gene-therapy trials that will help further research into this condition and hopefully provide a possible cure in the future [21].

## 4. Discussion

As information on human genetic disorders increases, it is clear that inherited ocular conditions have significant genetic heterogeneity [2]. As such, the diagnostic odyssey for these patients can take upwards of several years, with multiple medical care providers and diagnostic tests [2]. The creation of the MOGC improves healthcare delivery along three dimensions: in clinical care, for patients to have streamlined appointments with multiple providers who communicate directly in a single location; in research, as patients who underwent testing contribute to gene discovery, clinical phenotyping, and biobanking; and in education, to expose medical trainees of all types to these rare diagnoses. All of these help provide extensive and comprehensive ophthalmic genetic care to patients in need.

A challenge in ophthalmic genetics”care’Is the heterogeneity in genetic diagnostic rate, with very low diagnostic rates for certain conditions. One way to address this challenge without a specialized clinic is to create a targeted panel that tests for all ophthalmic conditions. The Oculome sequencing panel is an example of one such test [14]. This panel was developed to screen for over 420 pediatric genetic ocular diseases, many of which overlap with those seen in the MOGC (e.g., ASD, MAC, CC, RD) [14]. A study assessing this panel demonstrated the diagnostic yield to be 24.5% (n = 68/277), with the solve rate depending on phenotypic subtype [14]. In comparison, the MOGC had diagnostic results in 33% of cases (n = 16/48), which includes syndromic conditions in addition to ocular diseases. This was largely due to the multidisciplinary medical and ophthalmic nature of the team, in addition to the variety of testing that was able to be offered to patients. This multidisciplinary approach allows for the identification of syndromic conditions that would otherwise be missed due to ophthalmic features that are often overlooked, such as the confirmed genetic diagnosis of Axenfeld–Rieger in a family with phenotypic spectrum severity or the conclusion of a long diagnostic journey for the patient with Bosch–Boonstra Optic Atrophy. The patient with *RPGRIP1*-related LCA was appropriately directed towards resources such as options for possible gene therapy. For the pediatric ophthalmologists managing many of these patients’ primary ocular care, having a diagnosis improves the ability to counsel patients on prognosis regarding future vision, while additional test results help with clinical management. As illustrated in the case reports highlighted above, the MOGC’s multidisciplinary approach allows for increased research into rare conditions, similar rates of diagnosis, and better outcomes for patients.

Advances in genetic testing over the last decade have helped improve diagnostic capacity for genetic disorders with a scope spanning from narrow tests such as single-gene targeted testing and gene panels to broad tests such as exomes and genomes [17]. In reality, there are practical considerations between panel-based and exome sequencing. In the MOGC experience, panel tests have a 3–4-week turnaround time and are frequently covered by insurance, while exome sequencing has a turnaround time of 3–4 months for results and a higher rate of insurance denial, leading to higher out-of-pocket costs [22,23]. Our team at MOGC works together to efficiently utilize genetic testing following evaluation by all providers, especially since clinical features vary significantly based on the affected gene, which can impact gene-specific management plans [15]. For example, in a study assessing phenotypic variability among 128 individuals with clinical Axenfeld–Rieger Syndrome, Reis and colleagues examined causative variants in *PITX2* and *FOXC1* gene-coding regions via Sanger sequencing and/or research-based exome sequencing [15]. Exome sequencing was completed in 41% (n = 24/59) of *PITX2* and 57% (n = 39/69) of *FOXC1* probands [15]. Patients seen in the MOGC for a diagnosis of anterior segment dysgenesis (ASD) were similarly recommended to undergo targeted testing based on their phenotype prior to completing broad testing such as whole-exome sequencing. Fifty-seven percent of patients with ASD who completed testing were identified to have a diagnostic variant following this approach (half with targeted-only testing, half with a combination targeted to reflex broad testing). Methods to evaluate the genetic cause of Mendelian forms of cataract also exist, such as Cat-Map, an online chromosome map and reference database for inherited (and age-related) forms of cataract [16]. In a clinical setting, it is useful to differentiate between “syndromic” and “non-syndromic” forms of cataract based on the presence of nonocular anomalies in a patient, as the genotypes are distinct and can inform testing approach [16]. In the MOGC, for a referral diagnosis of congenital cataract, 64% of patients had an ocular-only presentation (n = 9/14) and were counseled to undergo specific gene panel testing initially. Of those with an ocular-only presentation who completed genetic testing (n = 6/9), 50% (n = 3/6) had a diagnostic test result. Being able to complete targeted testing for patients and then broaden if testing is nondiagnostic allows for lower use of healthcare resources (fewer patients needing insurance authorization), and a more rapid turnaround time for patients who receive a genetic diagnosis on initial testing [13,22,23,24].

Administratively, there are numerous advantages to the MOGC that can also be characterized. The ophthalmic geneticist works in conjunction with the medical geneticist and genetic counselor on testing strategies (selecting correct panels, interpreting test results). The medical geneticist can give input on dysmorphology, syndromic differential diagnoses, and metabolic testing for systemic phenotyping. The genetic counselor educates the patient on testing options, result implications, and explains testing results. Patients can also be enrolled in research studies for gene discovery, genotype–phenotype correlations, and treatment studies from the in-house research team. This team structure is similar to other Ocular Genetics Programs such as the one established at the Hospital for Sick Children in Toronto, which in its first decade saw more than 6000 families [1]. Data collected by that clinic showed that upwards of 95% of patients not only were satisfied with their visit, but would recommend the clinic to others primarily due to the ease in having a single appointment with both the medical genetic and ophthalmic genetic providers [1]. The importance of same-day pretest genetic counseling to discuss implications of testing cannot be stressed enough, maybe even more so for those patients who defer genetic testing, as this counseling gives them the ability to make their decision following clear education on what it will mean for them and their care.

The multidisciplinary approach provides patient-centered care and may lead to faster diagnoses given the ability to address both ocular-only and syndromic presentations. There are certainly challenges to the MOGC that require further study. For example, the care model is time- and resource-intensive with no reward in the traditional fee-for-service reimbursement system, which can limit provider availability. There is also a long wait time for appointments as the clinic is referral-based. Once patients are in the clinic, they can choose not to undergo genetic testing or, as seen in 38% of patients in this category, there may be an issue getting the DNA sample/testing kit returned. This barrier could be addressed by having patients draw blood or do the buccal swab/saliva test directly at the visit. Uncertain genetic testing results (variants and genes of uncertain significance) may limit diagnostic rate, but also present an opportunity for the better classification of genetic variants and research efforts to establish gene–disease relationships. The lower diagnostic yield for specific conditions seen in clinic compared to those reported in the literature could be attributed to a more complicated patient mix, as some of the cases were referred by other physicians who had reached the end of possibilities for testing. We have the opportunity for gene discovery research or functional validation in animal models or patient-derived organoid models to help clarify the definitive disease cause. Ultimately, the creation and maturation of more interdisciplinary clinics will improve the generation of specific testing results that can contribute to clinical genetic research.

## 5. Conclusions

The study goal was to evaluate whether our MOGC provides advantages to patients with inherited ocular conditions. The benefits of the clinic for patients outweigh administrative and systemic hurdles. We have found that a multidisciplinary approach has not only helped solve diagnostic odysseys, but has also helped discover new genetic conditions. We are motivated to share our experience in creating this program with other academic institutions so that we may develop a preferred practice pattern and improve diagnosis, risk prediction, drug development, and overall patient care and satisfaction in this population. Further research is planned to assess the patient experience and improve quality of care delivery.

## Figures and Tables

**Table 1:** Diagnostic test results for MOGC referral conditions with values > 10%; note that any results which are “pending” at the time of this manuscript were excluded (n = 4).

Referral Condition	Diagnostic Yield, % (n)
Anterior-Segment Dysgenesis (ASD)	57% (n = 4/7)
Nystagmus (N)	33% (n = 2/6)
Optic Nerve Anomaly/Atrophy (ONA)	30% (n = 3/10)
Congenital or Juvenile Cataract (CC)	36% (n = 4/11)
Retinal Dystrophy (RD)	20% (n = 1/5)
Microphthalmia/Anophthalmia/Coloboma (MAC)	13% (n = 1/8)
Corneal Dystrophy (CD)	0% (n = 0/2)

## Data Availability

Not applicable.
